# Increased phage resistance through lysogenic conversion accompanying emergence of monophasic *Salmonella* Typhimurium ST34 pandemic strain

**DOI:** 10.1099/mgen.0.000897

**Published:** 2022-11-16

**Authors:** Oliver J. Charity, Luke Acton, Matt Bawn, Eleonora Tassinari, Gaёtan Thilliez, Marie A. Chattaway, Timothy J. Dallman, Liljana Petrovska, Robert A. Kingsley

**Affiliations:** ^1^​ Quadram Institute Bioscience, Norwich Research Park, Norwich, NR4 7UQ, UK; ^2^​ University of East Anglia, Norwich NR4 7TJ, UK; ^3^​ Earlham Institute, Norwich, NR4 7UZ, UK; ^4^​ Gastrointestinal Bacteria Reference Unit, UK Health Security Agency (UKHSA), London, NW9 5EQ, UK; ^5^​ Animal & Plant Health Agency (APHA), Weybridge, London, KT15 3NB, UK

**Keywords:** epidemiology, evolution, genomics, phage, monophasic, salmonella

## Abstract

*
Salmonella enterica* serovar Typhimurium (*S*. Typhimurium) comprises a group of closely related human and animal pathogens that account for a large proportion of all *Salmonella* infections globally. The epidemiological record of *S*. Typhimurium in Europe is characterized by successive waves of dominant clones, each prevailing for approximately 10–15 years before replacement. Succession of epidemic clones may represent a moving target for interventions aimed at controlling the spread and impact of this pathogen on human and animal health. Here, we investigate the relationship of phage sensitivity and population structure of *S*. Typhimurium using data from the Anderson phage typing scheme. We observed greater resistance to phage predation of epidemic clones circulating in livestock over the past decades compared to variants with a restricted host range implicating increased resistance to phage in the emergence of epidemic clones of particular importance to human health. Emergence of monophasic *S*. Typhimurium ST34, the most recent dominant multidrug-resistant clone, was accompanied by increased resistance to phage predation during clonal expansion, in part by the acquisition of the mTmII prophage that may have contributed to the fitness of the strains that replaced ancestors lacking this prophage.

## Data Summary

Three collections of whole-genome sequences of strains were used in the study and accession numbers and associated metadata can be found in Supplementary Tables as indicated. The first was a collection of 109 strains for which phage type were known, that were a subset of 134 strains comprising 2–5 randomly selected isolates of common phage types isolated from human clinical infections or animals in the UK [1] (Table S1, available in the online version of this article). The second collection comprised all 1413 whole-genome sequences of *S*. Typhimurium or 1,4,[5],12:i:1,- (monophasic *S*. Typhimurium) isolated from human infections by UKHSA between April 2014 and October 2015 [2] (Table S2). The third collection comprised 439 whole-genome sequences from the Short Read Archive (SRA) of monophasic *S*. Typhimurium ST34 isolated between 1998–2019 from diverse geographical locations worldwide and was used to investigate the evolutionary history of prophage mTmII and construct a time-scaled phylogenetic tree to identify time of acquisition (Table S3).

**Impact Statement**

Phages are viruses that replicate through killing bacteria or residing as prophages integrated into bacterial genomes. Replication of phages results in killing of the bacterium that can have a significant impact on susceptible populations of bacteria. Phage-based therapies are a promising alternative to antibiotics, but little is known about the effect phages have on bacterial pathogen population structures. Like antibiotics, bacteria may become resistant to phage by many different mechanisms, including integration of phages into the genome. Phages also play an important role in the evolution of bacteria by mediating the transfer of bacterial DNA between bacteria, a process critical to adaptation and epidemic emergence of new strains. We have investigated the variation in sensitivity to phage predation of *Salmonella enterica* serovar Typhimurium (*S*. Typhimurium), a leading cause of food-borne illness globally. We found that strains of *S*. Typhimurium that are particularly successful at circulating in multiple livestock species, and consequently cause most infections in people, tend to be more resistant to phage. Furthermore, the emergence of new successful strains was accompanied by increasing resistance to predation by phage, including by integration of a new phage into its genome. We conclude that resistance to phage may be important for the spread within multiple host species but may limit further evolutionary adaptation.

## Introduction

Phages are the most abundant biological entity in the biosphere [3], yet comparatively little is known about their impact on bacterial populations. Bacteria constantly come into contact with phage in the host gut and external environment resulting in both a potentially beneficial and adverse impact on survival and evolution [4]. Phage predation occurs if the bacterial strain is sensitive to virulent infection resulting in destruction of the cell by lysis that may result in reduction in population size and potentially exclusion from a niche entirely. Alternatively, lysogenic conversion by temperate phage may result in resistance to related phage and in some cases acquisition of cargo genes affecting population dynamics [5], including genes involved in virulence, metabolism or antibiotic resistance genes [6–8], all of which have the potential to increase the fitness of the bacterial strain [2, 9, 10]. Phage play an important role in horizontal transfer of genetic material by specialized, generalized or lateral transduction [11]. Indeed, the frequency of horizontal transfer of chromosomal genes by lateral transduction may exceed that of other mobile genetic elements, and thereby represent a key mediator of microbial evolution [12].

Non-Typhoidal *Salmonella* (NTS) remains a significant global health risk and economic burden. An estimated 78 million human infections per year contribute to 4 million disability adjusted life years, more than any other food-borne pathogen or chemical agent [13]. Approximately a quarter of all NTS strains isolated from clinical infections in the UK are serovar Typhimurium (*S*. Typhimurium), second only to *S*. Enteritidis [14]. Controlling *S*. Typhimurium is complicated by its a genotypic diversity that is driven in part by host adaptation and anthropogenic selection [1]. The population structure of *S*. Typhimurium is composed of two distinct groups of lineages designated alpha (α) and beta (β). Clade α is composed of lineages associated with agricultural animals and dominant epidemic/pandemic clonal lineages, and β predominantly of lineages containing strains with a restricted host range, including wild avian species [1]. Consequently, the risk to human health from strains of *S*. Typhimurium from different lineages differs markedly, highlighting the importance of understanding the relationship of population structure and genotypic variation in risk assessment [1, 15]. Despite phage being the most abundant biological entity in the biosphere, little is known of their influence on this population structure.

Until recently, *S*. Typhimurium has been sub-typed in the UK and elsewhere using the Anderson phage typing scheme that uses differential sensitivity to a panel of 30 phage preparations to distinguish strains [16]. Currently, 207 definitive types (DTs) are recognized but other common phage sensitivity profiles may also be assigned untypable (U), for example U288 [17]. Temporally, and in some cases geographically, clustered groups of isolates of a single phage type has been used to infer clonal relationship for outbreak detection [18, 19]. However, the relationship between phage type and phylogeny are not well understood. For example, phage-type variation within a clonal group of *S*. Typhimurium due to phage-type switching is observed, forming a phage-type complex. Phage types can also be polyphyletic, where the same phage types are present in distinct clonal groups [1]. DT193 that is defined by resistance to all 30 phage preparations in the Anderson scheme is particularly common in multiple clades likely due to saturation of the typing scheme [1, 18, 20–22].

Since public health surveillance of *S. enterica* clones began during the 1960s, it has become apparent that *S*. Typhimurium epidemiology is characterized by emergence, clonal expansion, decline, and replacement of successive dominant multidrug-resistant epidemic or pandemic clones [23]. Four successive dominant clones of *S*. Typhimurium since the year 1960 have been characterized by phage types DT9, DT204 complex, DT104, and currently DT193/DT120 complex, respectively [1]. Each of these *S*. Typhimurium clones exhibit multidrug resistance, typically including resistance to ampicillin, sulfonamides, streptomycin and tetracycline [23]. The drivers of the emergence and replacement of dominant clones is not fully understood but is not likely due to selection for antibiotic resistance or immune evasion since each of the clones encode similar resistance profiles and share immune-dominant antigens [24–26]. For the currently dominant MDR strain (monophasic *S*. Typhimurium ST34), which is monophasic due to a deletion in the phase 2 flagellin gene *fljA* and predominantly DT193/DT120, some potential drivers of emergence have been proposed. This includes acquisition of transferable copper resistance encoded on an integrative conjugative element (SGI-4) and the *sopE* virulence gene on the mTmV prophage that may provide a fitness advantage in livestock in which copper supplementation of feed is now common [2, 27, 28].

Coevolution of bacteria and phage has been well documented *in vitro* [29], but their dynamics in nature have proven more difficult to study. The availability of the whole-genome sequence of bacterial pathogens revealed the effect of gene flux on phage predation and population structure. Phage sensitivity of Shiga toxigenic *Escherichia coli* was reported to be stable within sub-lineages with altered sensitivity variants only outcompeting the prevailing sensitivity profile clones if accompanied by a competitive advantage [9]. We investigated the relationship between phage type and phylogeny of *S*. Typhimurium and infer the consequences of the predator–prey relationship on the evolution of this pathogen. To address whether host-restricted or dominant epidemic clones differ in their resistance to phage predation we compared phage sensitivity of strains from subclades of *S*. Typhimurium with well-characterized epidemiology. Finally, we investigated changes in phage sensitivity and the molecular basis of phage resistance during the emergence of monophasic *S*. Typhimurium ST34, the currently dominant multi-drug-resistant strain in the UK and the European Union.

## Methods

### Strain selection

For analysis of phage sensitivity within *S*. Typhimurium three collections of whole-genome sequences of strains for which phage-type data were available were used. The first comprised sequence of 109 strains that were a subset, for which phage type was known, of 134 strains that contained two–five randomly selected strains of common phage types isolated from human clinical infections (from UK Health Security Agency surveillance) or animals (from Animal and Plants Health Agency surveillance) in the UK [1] (Table S1). A second collection comprised all 1413 whole-genome sequences of *S*. Typhimurium or 1,4,[5],12:i:1,- (monophasic *S*. Typhimurium) isolated from human infections by UKHSA between April 2014 and October 2015 [2], of which 723 were in the monophasic *S*. Typhimurium ST34 epidemic clade (Table S2). A third collection of 439 whole-genome sequences from the Short Read Archive (SRA) of monophasic *S*. Typhimurium ST34 isolated between 1998–2019 from diverse geographical locations worldwide were used to investigate the evolutionary history of prophage mTmII and in construction of time-scaled phylogenetic tree to identify time of acquisition (Table S3).

### Bacterial culture and strain construction

*S*. Typhimurium was routinely cultured in Lysogeny broth (LB; 1 % tryptone, 1 % NaCl, 0.5 % yeast extract in ddH_2_O) or on LB plates containing 1.5 % agar supplemented with 30 µg ml^−1^ nalidixic acid, 50 µg ml^−1^ kanamycin or 25 µg ml^−1^ chloramphenicol as appropriate. Allelic exchange was undertaken using the method described by Datsenko and Wanner [30], with primers shown (Table S4). Briefly, 50 nucleotides upstream of the ATG start codon and downstream of the non-sense codon were included at the 5′ end of oligonucleotides used for amplification of the *aphII* gene from plasmid pKD4, or the *cat* gene from pKD3. Allelic exchange was carried out in *S*. Typhimurium strains containing pSIM18 [31]. For construction of mTmII::*cat* lysogens of *S*. Typhimurium DT120 strains, the *cat* gene was first introduced into an intergenic region upstream of the ORF1 of prophage mTmII in *S*. Typhimurium ST34 strain S04698-09 (S04698-09::mTmII::*cat*). The prophage mTmII::*cat* was then transferred to a phage-cured strain of *S*. Typhimurium (ΔΦ4/74) [32], that was further modified by introduction of an *aphII* gene in the intergenic region upstream of the *iciA* gene (strain ΔΦ4/74::‘*iciA::aphII*), to enable selection of prophage mTmII::*cat* lysogens. For transfer of prophage mTmII::*cat*, the donor strain S04698-09::mTmII::*cat* and recipient ΔΦ4/74::‘*iciA::aphII* were co-cultured in LB broth for 18 h at 37 °C with shaking, and serial 10-fold dilutions plated on LB agar plates supplemented with 30 µg ml^−1^ chloramphenicol and 50 µg ml^−1^ kanamycin to select prophage mTmII::*cat* lysogenic transductants (ΔΦ4/74::mTmII::*cat*). Prophage mTmII was then introduced into *S*. Typhimurium DT120 strains L00745-07 and L00979-07 that naturally encode tetracycline resistance on the *tetACR* locus [28], by co-culture with the ΔΦ4/74::mTmII::*cat* strain, with prophage mTmII::*cat* lysogenic DT120 strains selected on LB agar plates supplemented with 30 µg ml^−1^ chloramphenicol and 5 µg ml^−1^ of tetracycline (L00745-07::mTmII::*cat* and L00979-07::mTmII::*cat*). The desired genotype of recombinant strains was confirmed by PCR amplification using oligonucleotides that primed the reaction by annealing to sites flanking the targeted locus [33].

### Determination of phage type of *S*. Typhimurium isolates and phage sensitivity

Phage typing was carried out at Public Health England (PHE) and the Animal and Plant Health Agency (APHA) using the PHE phage typing protocol for *Salmonellae* and *Shigella flexneri* as part of routine surveillance based on the scheme described previously [17]. Determination of phage sensitivity to selected phage from the Anderson scheme of strains was undertaken in the lab using a similar method. Bacterial strains were cultured on LB agar plates to form single colonies. A single colony was used to inoculate 5 ml of LB broth and incubated static at 37 °C for 2 h. An LB agar plate was flooded with the culture and left to dry for 1 h. Once dried, 10 µl of each phage suspension containing approximately 100 plaque forming units was spotted onto the plate and incubated for 4–16 h. Plaque morphology was identified visually, with reference to the phage-typing scheme.

### Quantifying bacterial resistance to phage

A phage resistance index (*Ri*) of an isolate was calculated based on the phage type from the Anderson phage-typing scheme where *Ri* refers to the resulting number between 0 and 1 that is equal to the inverse fraction of phage preparations that resulted in lysis of the isolate during culture in overlay agar assays. To quantify the effect of prophage mTmII lysogeny on phage sensitivity we determined the optical density of cultures of *S*. Typhimurium DT193 strains S04332-09 and S04689-09 that are prophage mTmII lysogens, DT120 strains L00745-07 and L00979-07 that lack prophage mTmII, and DT120 strains L00745-07::mTmII::*cat* and L00979-07::mTmII::*cat*, in the presence or absence of Anderson typing scheme phage 8, 18 and 29. *S*. Typhimurium strains and phage were cultured with a multiplicity of infection of 1 in atmospheric conditions at 37 °C with shaking for 24 h in a Bioscreen C MGR, reading the optical density at 600 nm every 15 min. The area under the curve was determined using the definite integral from 5 to 15 h with R package DescTools [34]. The Mann–Whitney–Wilcoxon test was used to assess statistical significance.

### Whole-genome sequence data analysis

For comparative genome analysis and mapping of K-mers, complete and closed whole-genome sequence of *S*. Typhimurium strain S01569-10 (DT120) or strain S04698-09 (DT193) were used as reference strains as indicated in the text and legends [2]. Draft genome assemblies were generated using de Bruijn graph-based SPAdes v-3.10.1 [35], with K-mer lengths of 31, 41 and, 51, before annotation using Prokka v-1.11 [35–37]. Comparative genetic diagrams were constructed using R package genoplotR [38]. SNPs were identified in WGSs by aligning reads using BWA-MEM [39], variant calling with Freebayes [40] and SNP filtering using vcflib/vcftools [41], combined as a pipeline using Snippy v4.3.6 [42] with reference to the complete and assembled whole-genome sequence of strain *S*. Typhimurium strain S04698-09 [28]. Determination of gene presence in short-read sequence data generated using Illumina sequencing was by mapping and local assembly of reads using ARIBA with custom databases [43]. Gene presence was defined as sequence with >90 % nucleotide sequence identity to the reference over 80 % of the target gene. The presence of prophage mTmII was identified through mapping sequence reads to a custom database comprising each gene of prophage mTmII in ST34 strain S04698-09 [28] with presence defined as >90 % nucleotide sequence identity to >58 of the 63 prophage mTmII genes to account for small deletions present in some of the prophage.

### Phylogenetic reconstruction

Potential regions of recombination were removed from core-genome alignments using Gubbins v2.3.4 [44]. RAxML was employed to construct phylogenetic trees in this study from core-genome SNPs [45], using the general-time reversable model of nucleotide substitution, with gamma distribution for amongst-site rate variation (GTR), typically with 1000 bootstraps [46]. Using the gamma distribution for amongst-site rate variation allowed for better comparison between phylogenetic trees. ModelFinder was used to determine if different models of nucleotide substitution constructed phylogenies with significantly improved log-likelihoods, or fundamentally different topology, as implemented in the IQtree pipeline [47, 48]. For the data in this study all phylogenies were constructed using RAxML [49] with the GTR and gamma model of amongst-site rate variation, and there were no significantly improved models when considering other models of nucleotide substitution or rate variation. Second order clades within the population structure were determined using rhierbaps [50].

### Ancestral state estimation

Maximum-likelihood- (ML) based states at each hypothetical ancestral node were estimated with Ancestral Character Estimation (ACE) from R package ape [51] and pastML [52], using maximum posterior probabilities. The transition rate (*Q*) matrices were estimated and models allowing different rates of transition were used. A Markov chain with Monte-Carlo sampling (MCMC) approach was used with discrete character mapping using posterior sampled maps from SIMMAP, and the results plotted as probability density across branches of fixed ML phylogenetic trees, generated as previously described [53]. One thousand sampled stochastic character maps were constructed after a burn-in period of 1000 iterations for *Q,* followed by 1 000 000 Markov-chain steps, and sampling for the posterior every 1000 generations using the pre-computed distribution of *Q* [54]. Competing statistical models using either equal rates of transition (*Q*) or allowing different values for *Q* were compared using the likelihood ratio test (LRT) such that the more complex model to reside in 5 % of the right-hand tail of a χ^2^ distribution with one degree of freedom was considered significant. For MCMC calculated distributions, this was conducted using mean log-likelihoods of sampled estimated histories from MCMC analysis using both an equal rate *Q*, and pre-computed distribution of values of different rates for *Q* with the starting value estimated from the data. Resulting data was interpreted and viewed using R package phytools [54], iTOL [55] and pastML [52]. To test for further evidence that the phenotype of the root node was DT120 based on the outcome of ML estimation, probabilistic MCMC analyses was undertaken as described above. To assess the probability of DT120 occurring at nodes toward the root branches and estimate the probability that it is the root node phenotype tips of the phylogeny were considered as either of two states, ‘DT120’ or ‘other phage type’. To estimate the probability that the root node was DT193 tips were considered as one of two states, ‘DT193’ or ‘other phage type’, and MCMC analysis carried out as described above.

### Bacterial genome-wide association

Genome-wide association was conducted to discern potential genetic polymorphisms associated with DT193, considering this as a trait of increased phage resistance, using 489 DT193 isolates and 108 DT120 isolates (Table S2). This was undertaken through DNA of length K (K-mer) based analysis. The population structure was estimated using genome hash-based Mash, which generated a three-dimensional distance matrix [56]. K-mers were mined from WGS assemblies using frequency-based string mining algorithm FSM-lite [57]. Subsequently, a mixed-linear model approach was used for testing K-mer significance, implemented with sequence element enrichment (SEER) [58]. This was initially conducted with no significance filtering, but a subsequent K-mer filtering significance level was established by keeping the top 1 % of K-mers in the range of -log_10_ *P*-values, which determined an LRT *P*-value threshold for K-mer significance of 1×10^−13^. K-mers above an LRT *p*-value of 1×10^−6^ were plotted.

### Quantification of clonal expansion using nearest-neighbour patristic distances

In order to quantify clonality and infer a measurement for clonal expansion, the pairwise core-genome SNP distance between tips (patristic distance) of a core genome SNP constructed phylogeny (the co-phenetic matrix) was extracted using R package Ape [59]. The pairwise SNP distance of the closest relative of each taxon was extracted, using this data to measure differences of within-clade patristic distances. A clade with high patristic distance indicated that many SNPs have occurred between the isolates, increasing the branch lengths between them. A clade with low patristic distance will have fewer SNPs between them, indicating more frequent sampling of a lineage, and increased clonal expansion.

### Temporally structured phylogenetics for prophage mTmII acquisition time

Acquisition of mTmII was determined through a temporally structured phylogenetic tree as determined by Bactdating [60]. The phylogenetic tree was rooted using *S*. Typhimurium reference SL1344 and the reference removed before undertaking 1 000 000 iterations of MCMC. To address whether the dates of ancestral nodes were being calculated based on the phylogenetic tree structure and not occurring by chance from the data, 50 permutation tests were undertaken and the distribution of the resulting 50 root-node dates assessed. All root nodes were further back in time than the estimate of the real data (6000bc – 1950) consistent with the estimate from the real data not being dependant on the associated isolation year data alone.

### Identification of prophage mTmII-related phages

ORFs from the nucleotide coding sequence of mTmII from reference monophasic *S*. Typhimurium ST34 were translated into amino acid sequences and subject to neighbour-joining phylogenetic reconstruction with phage sequences using VipTree and the VipTree phage database [61]. The nucleotide sequence of phages that clustered with mTmII and had >75 % sequence identity to mTmII as determined by VipTree were downloaded from the NCBI genome database and ORFs annotated by searching pFam-11.1 [62] using Hmmer-3.2.2 [63], and the NCBI non-redundant amino acid database using blastp. Annotated sequences were then plotted using genoPlotR [38] and associated with the amino acid sequence-constructed neighbour-joining tree.

## Results

### Resistance to all standard typing phages is the most frequent lineage profile within the *S*. Typhimurium population

The distribution of phage types in the population structure of *S*. Typhimurium was initially investigated in 1413 isolates from human infections in the UK between 2014–2015. A phylogenetic tree was constructed using 16 681 recombination-purged core genome SNP sites and 36 third-order clades that corresponded to subclades resulting from recent clonal expansion of epidemic clones were identified (Fig. S1A). Phage type of isolates within each subclade varied considerably (Fig. S1B). The proportion of isolates in each subclade that were of the most common phage type ranged from 13–100 %, with 28 of 36 subclades with greater than half of all isolates being of the most frequent phage type, and just five composed entirely of a single phage type. DT193 that is defined by resistance to all standard Anderson scheme-typing phage preparations was the dominant phage type in five clades, two more clades than any other phage type (Fig. S1C).

### Broad-host-range and livestock-associated lineages of *S*. Typhimurium exhibit greater resistance to phage

**Fig. 1.**
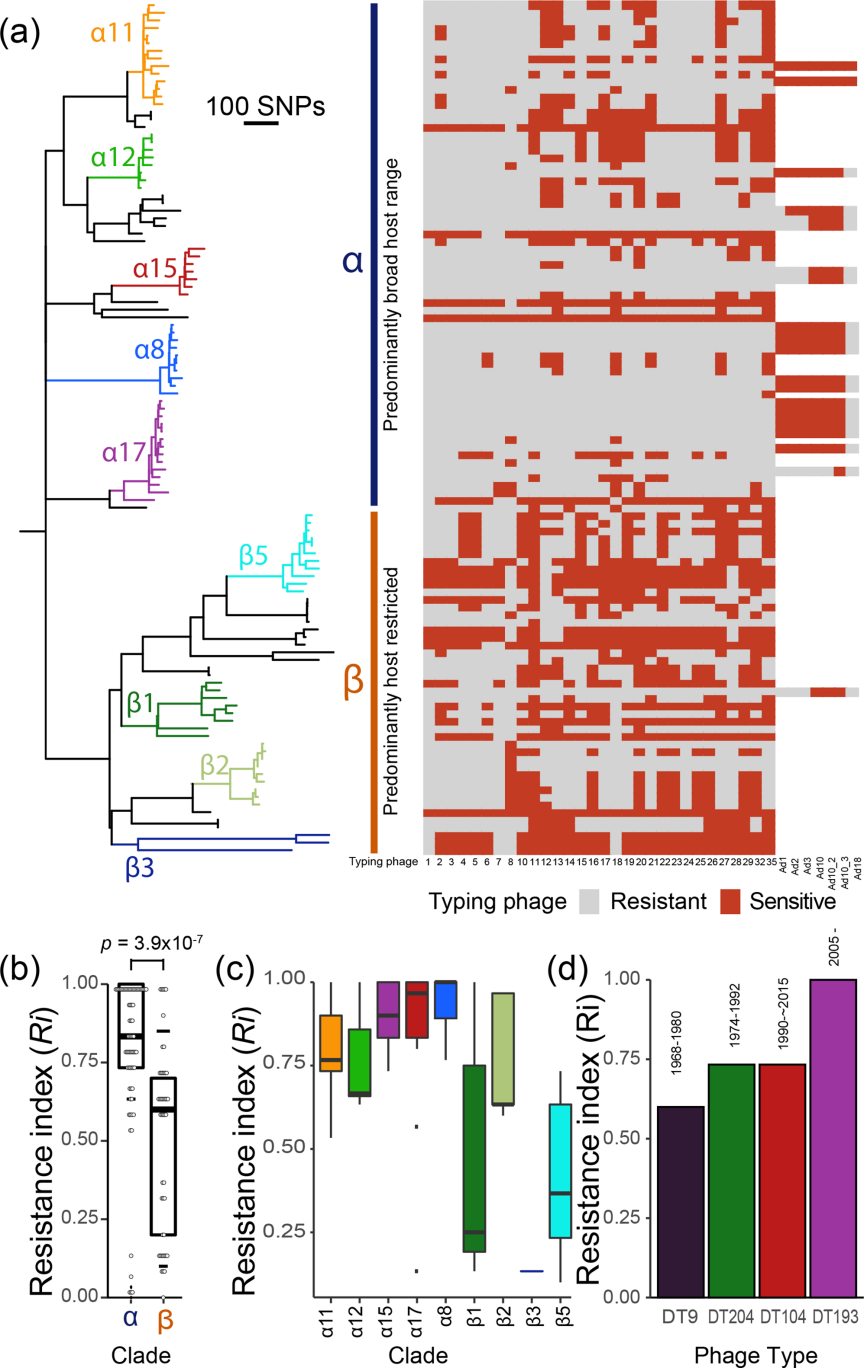
Variation in sensitivity to Anderson typing phage preparations within the population structure of *S*. Typhimurium. (a) Maximum-likelihood phylogenetic tree of 109 representative *S.* Typhimurium strains from human clinical infection or animals [1] constructed using 17 823 recombination-purged core genome SNP sites. Major lineages/clades are colour coded and the heatmap indicates sensitivity (red blocks) and resistance (grey blocks) to phage preparations from the Anderson phage typing scheme (standard phage 1, 2, 3, 4, 5, 6, 7, 8, 10, 11, 12, 13, 14, 15, 16, 17, 18, 19, 20, 21, 22, 23, 24, 25, 26, 27, 28, 29, 32, 35, and additional phage Ad1, Ad2, Ad3, Ad10, Ad10.2, Ad10.3, Ad18). First-order clade α (blue vertical bar) and β (orange vertical bar) are indicated. (b) Quantification of phage sensitivity (resistance index; *Ri*) of each strain (black dots) with mean (horizontal line), first and third quartiles (box), and range (vertical line) grouped by first-order clade α and β. (c) Quantification of phage sensitivity of each strain as grouped by third-order lineage with mean (horizontal line), first and third quartiles (box), range (vertical line), with box colours corresponding in (a). (d) Quantification of phage sensitivity (resistance index; *Ri*) (bars) of successive, dominant, epidemic, MDR clones of *S.* Typhimurium.

To address whether lineages of *S*. Typhimurium with distinct host range exhibited differential sensitivity to phage, we associated corresponding Anderson phage-typing scheme data with a phylogenetic tree of 109 representative *S*. Typhimurium strains reconstructed from recombination-purged core-genome sequence variation (Fig. 1a). We determined the resistance index (*R_i_*) for strains in first-order clades (α and β) that predominantly contain broad host-range or host-restricted strains, respectively [1], (Fig. 1a). Clade α exhibited a significantly greater *R_i_* than clade β (*P=*3.9×10^−7^)(Fig. 1b), indicating greater phage resistance of broad-host-range livestock-associated lineages. Five lineages from clade α exhibited greater resistance to phage, with *R_i_* values from ~0.6–1, while clades containing strains exhibiting host-adaptation or host-restriction [1] exhibited lower mean *Ri* values (0.14–0.76) with duck-associated clade β2 being the only lineage from clade β with *R_i_* values in the upper-mid range (0.7–1) (Fig. 1c) [64]. The *R_i_* of four successive dominant clones (DT9, DT204, DT104, and monophasic *S*. Typhimurium ST34, predominantly DT193 and DT120) over the last 60 years in Europe, had a trend to greater or equal resistance to phage with each successive clone (Fig. 1d).

### Emergence of the monophasic *S.* Typhimurium ST34 epidemic clade was associated with increased resistance to phage predation

**Fig. 2.**
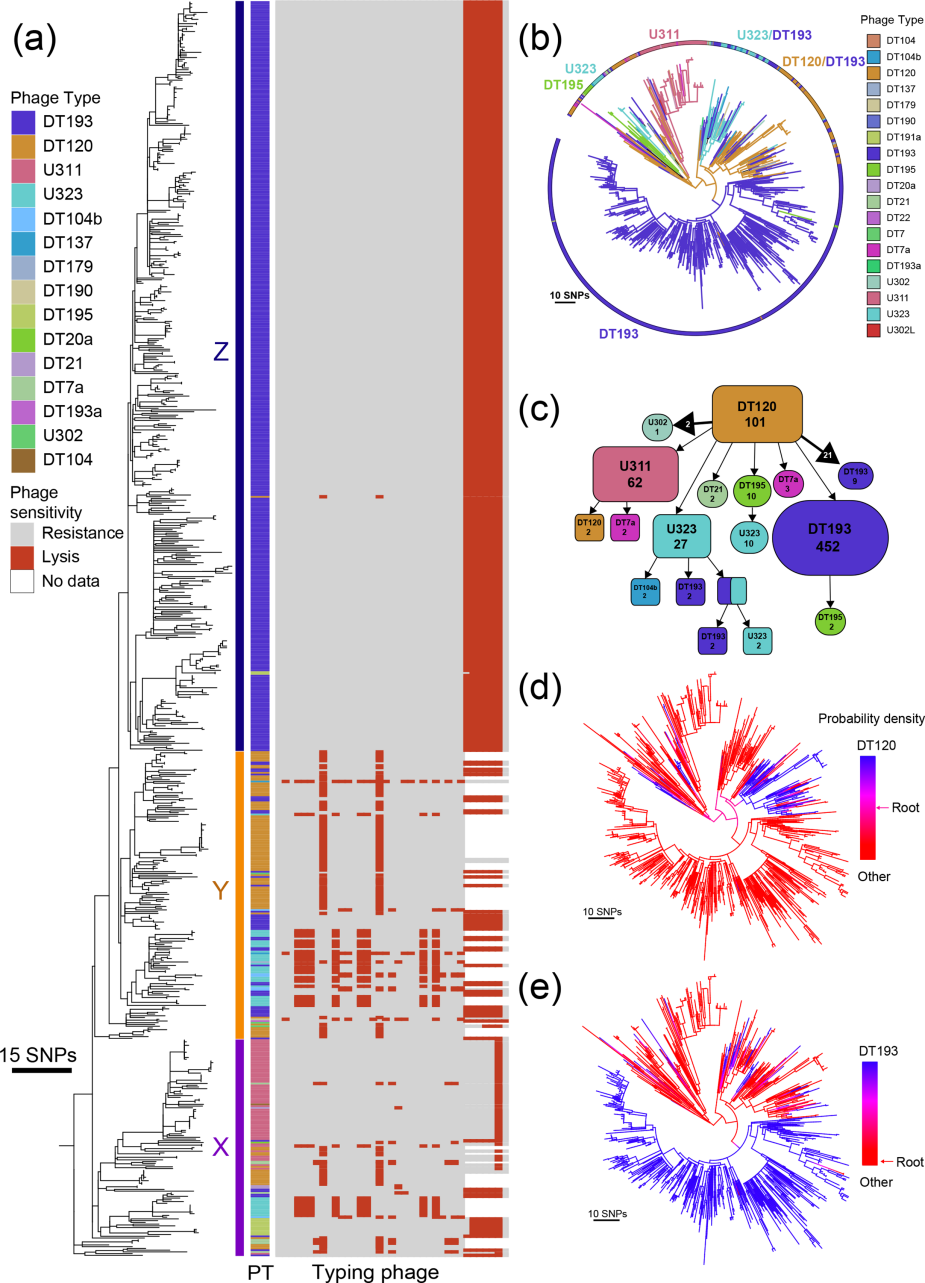
Variation in phage type and transitions between phage types during clonal expansion of monophasic *S.* Typhimurium ST34. (a) Maximum-likelihood phylogenetic tree constructed using 6403 recombination-purged core genome SNP sites from 723 monophasic *S.* Typhimurium isolates from human infections in the UK, 2014–2015. Corresponding phage sensitivity data is displayed in the heatmap. Subclades composed of primarily U311 isolates (x), predominantly DT120 and U311 isolates (y), and primarily DT193 isolates (z). (b) Maximum-posterior probability-based ancestral phage-type estimates inferred from the maximum-likelihood phylogenetic tree from (a). (c) Collapsed-branch node graph of the maximum-posterior probability-based ancestral phage type estimates from (b) displaying the number of tips that track back to a particular node (ancestor) without changes of phage type (numbers within shapes), and the number state changes between the two phage types that occur before tips (numbers in arrow heads). Lone tips with a single change in phage type have been excluded for ease of viewing. (d) Density map of the probability that each point in the history of the lineage had a phage type of DT120, with the corresponding probability of the root node being DT120 displayed on the key. (e) Density map of the probability that each point in the history of the lineage had a phage type of DT193, with the corresponding probability of the root node being DT193 displayed on the key.

We next investigated the dynamics of phage sensitivity during the emergence of monophasic *S*. Typhimurium ST34 lineage (clade α17 in Fig. 1, clade 12 in Fig. S1A), the most recently emerged dominant MDR clone. Isolates were predominantly DT193 or DT120 in this clade, although multiple other phage types have also been reported previously [28]. A maximum-likelihood tree of 723 strains of monophasic ST34 isolated by PHE from clinical infections for which phage-type data was available revealed a population structured with multiple subclades (*X*, *Y* and *Z* Fig. 2a). Proximally rooted subclades had longer terminal branches (*Y* and *Z*) than a large distal subclade (*X* in Fig. 2a). The 723 isolates were of 19 different phage types with the greatest phage-type diversity in the more basally rooted subclades (*X* and *Y* in Fig. 2a). The vast majority of isolates (94%) were of phage types DT193 (*n*=504), DT120 (*n*=108), U311 (*n*=63) and U323 (*n*=40). Isolates of each phage type were clustered within the population structure consistent with changes in sensitivity to phage followed by clonal expansion to different degrees. Ancestral state reconstruction using a maximum posterior probability (MPP) method, with transition rates estimated from the tip data, indicated that DT120 was the most likely root node ancestor, consistent with a relatively high frequency of DT120 isolates in multiple deeply rooted lineages radiating from the common ancestor (Fig. 2b). The dynamics of transition between phage types included 12 transitions to DT193, one giving rise to a population of 452 isolates in this dataset (Fig. 2c). Stochastic maps generated with MCMC using SIMMAP to test the probability of a DT120 and DT193 being the common ancestor at the root node indicated DT120 had the greatest probability at 47%, and a mean root node probability of 53 % for any of the other 18 phage types (Fig. 2d), while DT193 had a root node probability distribution of <5 % (Fig. 2e).

### Increased resistance to phage predation of monophasic *S.* Typhimurium ST34 was accompanied by increased clonal expansion

**Fig. 3.**
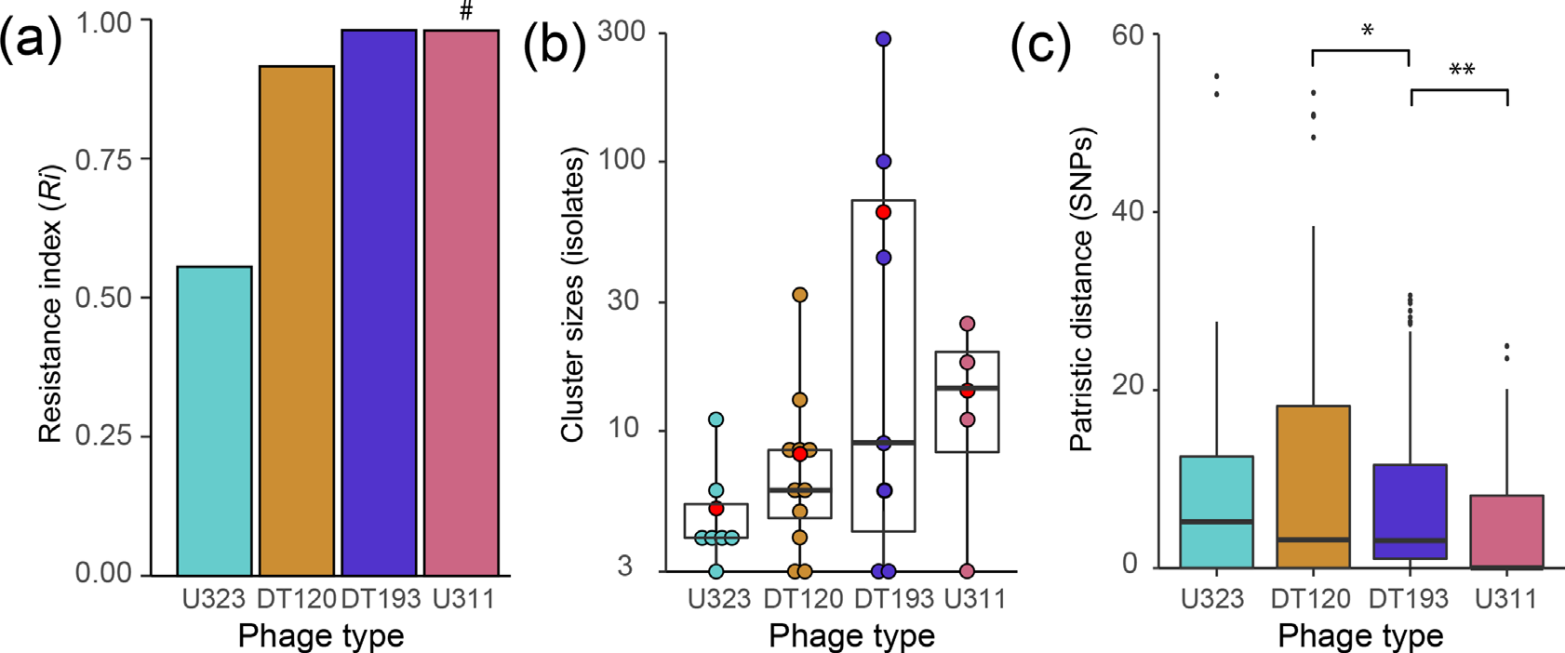
Analysis of the four most frequent phage types within monophasic *S.* Typhimurium ST34 lineage strains isolated from gastrointestinal infections in the UK, 2014–2015. (a) Phage resistance index (*Ri*) of each of the four most frequently observed phage types within strains of the monophasic *S.* Typhimurium ST34 lineage isolated from human gastrointestinal infections in the UK, 2014–2015. (b) Cluster sizes of each of the four most frequent phage types as defined by proximal tips that have the same phage type. Horizontal black lines indicate the median, and red filled circles indicate the mean. (c) Pairwise core-genome SNP distances (patristic distances) from subtrees of each of the four most frequent phage types. Horizontal black lines indicate the median. *= *P*<0.05, **= *P*<0.01. # U311 strains were resistant to seven additional typing phage preparations compared to DT193 strains but not reflected in the *Ri* reported here.

To investigate the impact of phage-type switches on sensitivity to phage, we determined the *R_i_* for each phage type represented in the monophasic *S.* Typhimurium ST34 clade. Resistance to phage increased in the order U323 (*R_i_* =0.567), DT120 (*R_i_* =0.967), DT193 (*R_i_* =1) and U311 (*R_i_* =1) (Fig. 3a). Although DT193 and U311 both had an *R_i_* of 1, the latter were resistant to lysis by seven additional phage preparations only used for typing of strains with the pan-resistant profile of DT193 to increase resolution. The dynamics of phage type transitions resulted in both decreased (DT120 to U323 and U311 to DT120) and increased (DT120 to DT193 or U311 and U323 to DT193) phage resistance (Fig. 2c). However, there were just two transitions that resulted in decreased resistance and at least 26 leading to increased resistance. Furthermore, the transition from DT120 to U323 later gave rise to three transitions to DT193, consistent with a general trend to increased resistance. Transitions to the phage types exhibiting the greatest level of phage resistance were also associated with increased clonal expansion. We reasoned that ST34 genotypes with increased fitness within the clonally expanding population would increase in relative abundance and would be present in clusters of closely related strains with low genetic diversity. Conversely, less successful genotypes would be expected to be under-sampled and be less closely related to their closest relative. In the collection of 723 ST34 isolates from England and Wales in a 19 month period in 2014–2015 the number of isolates in clusters of the same phage type increased with the resistance index (*Ri*) of the phage type (Fig. 3b). This was consistent with increased resistance imparting a fitness advantage. We next quantified the level of clonality of strains of each phage type by determining the number of SNPs that distinguished each strain compared to its nearest neighbour (patristic distance). The patristic distance exhibited an inverse relationship with the *Ri*, consistent with an increased clonal expansion (Fig. 3c).

### Phage type DT193 is associated with the presence of prophage mTmII

**Fig. 4.**
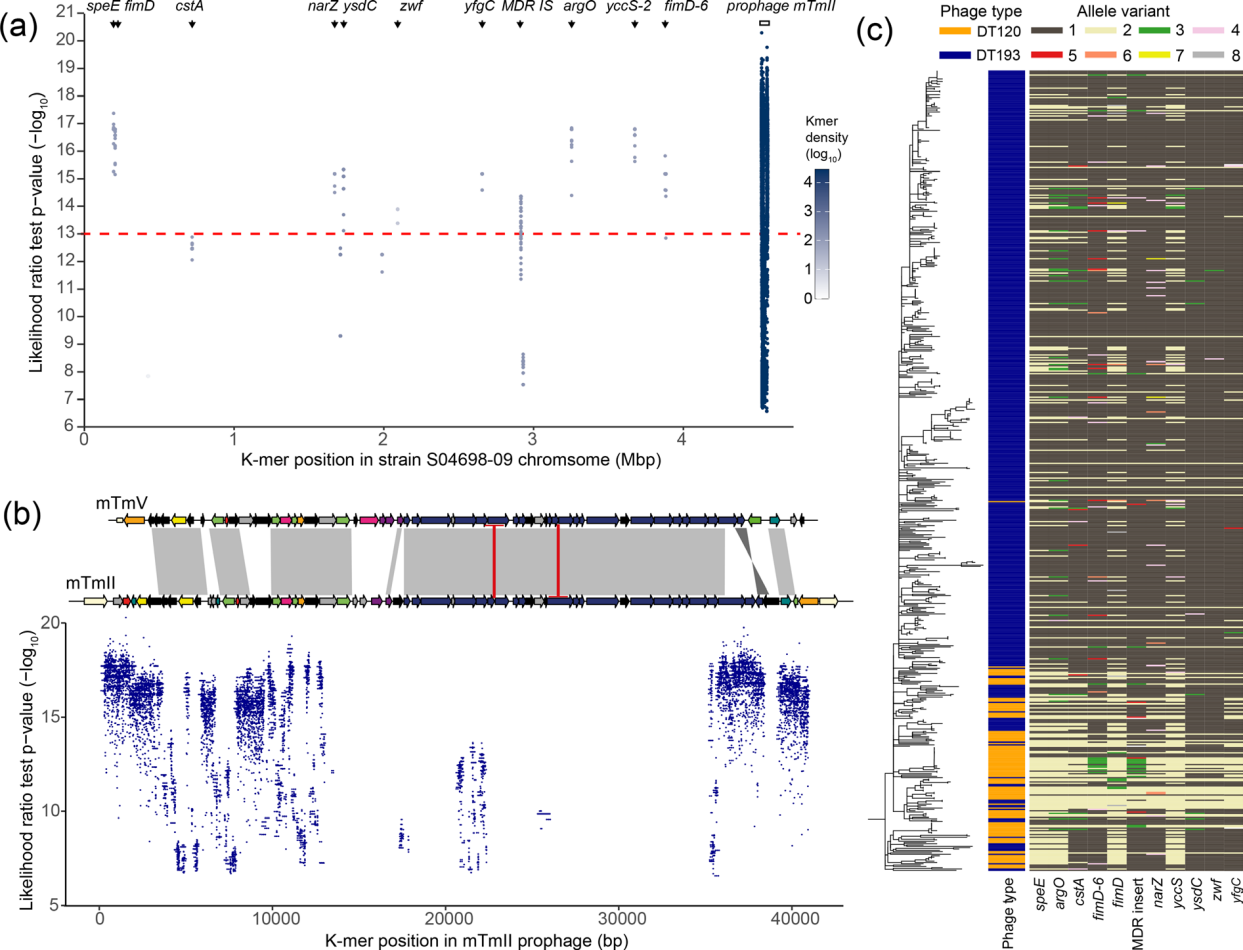
Bacterial genome-wide association (GWAS) of DT120 and DT193 strains from the monophasic *Salmonella* Typhimurium ST34 lineage (monophasic *S.* Typhimurium ST34). (a) Mapping locations of K-mers significantly associated with phage type DT193 to the genome of monophasic *S.* Typhimurium reference DT193 strain S04698-09 showing inferred likelihood ratio test (LRT) *P-*value cut-off (horizontal dashed red line). (b) Mapping locations of K-mers significantly associated with phage type DT193 to the nucleotide coding sequence of prophage mTmII. ORFs are colour coded by function as follows: hypothetical attB sites (white); integration and excision including IS elements (orange); phage lysogeny regulation (dark green); nucleases (yellow); phage defence (bright pink); virulence factor SopE (bright green); phage particle structure and assembly (dark blue); hypothetical toxin-antitoxins (red); host cell lysis (dark purple); phage associated hypothetical proteins (grey), and; other hypotheticals (black). (c) Distribution of allelic variants of genes corresponding to K-mers significantly associated with DT193 phage-sensitivity pattern (likelihood ratio test *P*-value<1×10^−13^) in the context of the population structure of a maximum-likelihood phylogenetic tree of 489 DT193 and 108 DT120 *S*. Typhimurium genomes.

Phage types DT120 and DT193 are the most common patterns of phage sensitivity in the ST34 epidemic clade and transitions between these phage types dominated the evolution of the clade during clonal expansion. To investigate the genomic changes associated with transitions from DT120 to DT193, we used a K-mer-based GWAS approach with 489 DT193 strains and 108 DT120 strains from the ST34 clade (Table S2). K-mers statistically associated with this transition (20 143 K-mers with LRT *P*<1×10^−6^) mapped to 12 locations of the monophasic S. Typhimurium ST34 strain S04698-09 genome (Fig. 4a). Genome sequence were within the *fimD* gene encoding a fimbrial usher protein, *speE* gene-encoding spermidine synthase, *cstA* gene-encoding carbon starvation protein, *narZ* gene encoding a nitrate reductase subunit, *ysdC* encoding a putative aminopeptidase, *zwf* encoding a glucose 6-phosphate to 6-phosphogluconolactone oxidase, *yfgC* encoding a peptidase, and a prophage previously termed prophage mTmII (Fig. 4d) [1]. More than 99.9 % of the K-mers (>10 000 k-mers) that were significantly associated with DT193 isolates mapped to prophage mTmII, with the other genomic locations exhibiting lower K-mer density.

### Prophage mTmII is related to *Shigella flexneri* prophages and lysogeny confers reduced sensitivity to some phage *in vitro*

Prophage mTmII consisted of 61 open reading frames (Table S5). Genes encoding phage capsid and assembly proteins had the greatest nucleotide sequence identity with *Shigella flexneri* phages, SfI, SfII, SfIV, SfV, and the monophasic *S*. Typhimurium ST34 prophage mTmV (Fig. S2) [2, 65]. Two genes with similarity to *yafO* and *ydaS* from *Escherichia coli* encode a possible toxin–antitoxin system [66, 67], notable since an unrelated toxin-antitoxin system, DarTG, has recently been shown to contribute to phage resistance [68]. No sequence with similarity to known O-antigen modification genes, commonly involved in increased resistance to phage super-infection, were present. Twenty genes of unknown function were also encoded on prophage mTmII, suggesting the mechanism affecting phage sensitivity may be novel.

**Fig. 5.**
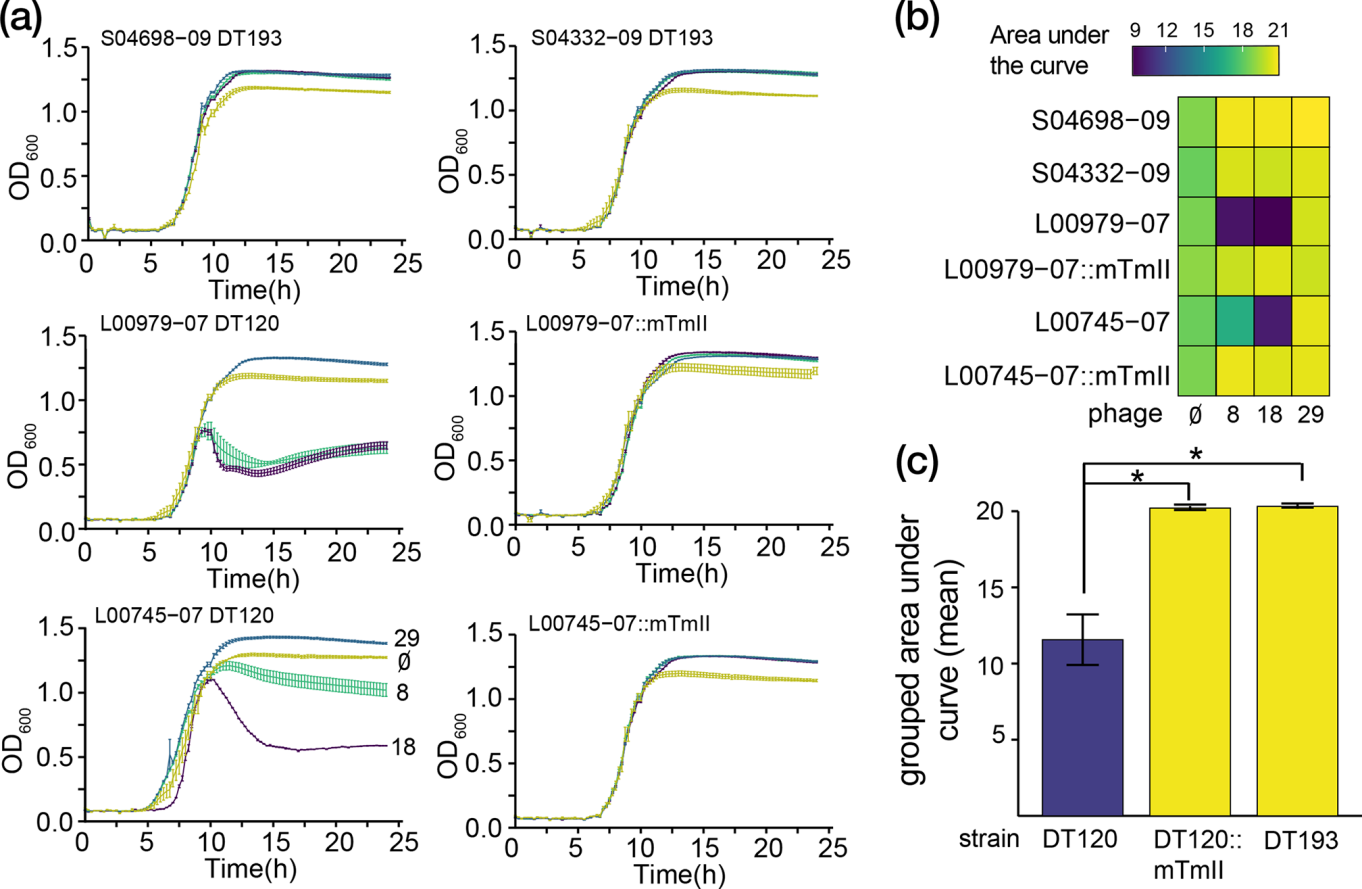
Prophage mTmII conveys reduced phage sensitivity to monophasic *S.* Typhimurium ST34 strains consistent with phage type DT193. (a) Growth of monophasic *S.* Typhimurium ST34 strains phage typed DT120 (L00745-07 and L00979-07), DT193 (S04698-09 and S04332-09), or DT120 strains with lysogenic prophage mTmII (L00745-07::mTmII and L00979-07::mTmII) without (yellow) and with Anderson-typing scheme phage preparations 29 (blue), 8 (turquoise) and 18 (dark blue) assessed through measuring the optical density (OD_600_) of cultures every 15 min. The mean and standard error of five replicates are displayed for each curve. (b) Area under the curve analysis of each curve from (a) quantified using the definite integrals from five to 15 h. (c) Area under the curve results for Anderson scheme phage preparations 8 and 18 grouped by phage type or lysogenic prophage mTmII. Mann–Whitney–Wilcoxon test results: *= *P*<0.05.

To test whether prophage mTmII lysogeny confers reduced sensitivity to typing phage ST34 DT193 strain prophage mTmII::*cat* was transferred to two DT120 isolates (strains L00979-07 and L00745-07) by co-culture *in vitro*. Co-culture of ST34 DT193 strain prophage mTmII::*cat* with ST34 DT120 strain L00979-07 or L00745-07 resulted in prophage mTmII lysogenic strains L00979-07 prophage mTmII::*cat* or L00745-07 prophage mTmII::*cat,* indicating that prophage mTmII is active and capable of transfer between strains. To investigate the impact of prophage mTmII lysogeny on sensitivity to typing-scheme phage preparations 8 and 18, *S*. Typhimurium parental and lysogen strains of DT120 were cultured in the presence of the phage and lysis measured by monitoring the OD_600_ of the culture. Wild-type DT120 strains L00979-07 and L00745-07 displayed a reduction in OD_600_ after 10 h of co-culture with phage 8 and 18, consistent with lysis by both phage (Fig. 5a). In contrast, DT120 strains L00979-07::mTmII::*cat* and L00745-07::mTmII::*cat* and wild-type DT193 strains S04698-09 and S04332-09 (Fig. 5b, c), exhibited no decrease in optical density compared to controls with no phage, consistent with prophage mTmII conferring resistance to these phages. DT120 strains lysogenic for prophage mTmII also had no significant difference (*P*=0.6857) in OD_600_ compared to wild-type DT193 strains when challenged with typing phage preparations 8 and 18, consistent with prophage mTmII determining phage type DT193 in monophasic ST34 strains and providing enhanced resistance to phage predation.

### Prophage mTmII was acquired multiple times in the monophasic *S*. Typhimurium ST34 population during the clonal expansion

To investigate the relationship of prophage mTmII acquisition during the evolution of monophasic *S*. Typhimurium ST34 its distribution was investigated in two collections of isolates. The first consisting of 723 ST34 isolates from human clinical infections in the UK from 2014 to 2015 (Table S2), and a second, including 374 monophasic *S*. Typhimurium ST34 isolated between 1998–2019 from diverse geographical locations worldwide (Table S3). The most parsimonious explanation for the distribution of prophage mTmII in the UK isolates was four independent acquisitions of prophage mTmII. Each one accompanied by a switch to DT193, consistent with prophage mTmII being a determinant of phage type DT193 (Fig. S3). In the more diverse collection of global ST34 isolates, the most parsimonious explanation for the distribution of prophage mTmII could be explained by eight independent acquisitions of prophage mTmII (Fig. S4). The patristic distance of strains with prophage mTmII was significantly lower (*P*<0.05) than those without prophage mTmII in the global strain collection.

**Fig. 6.**
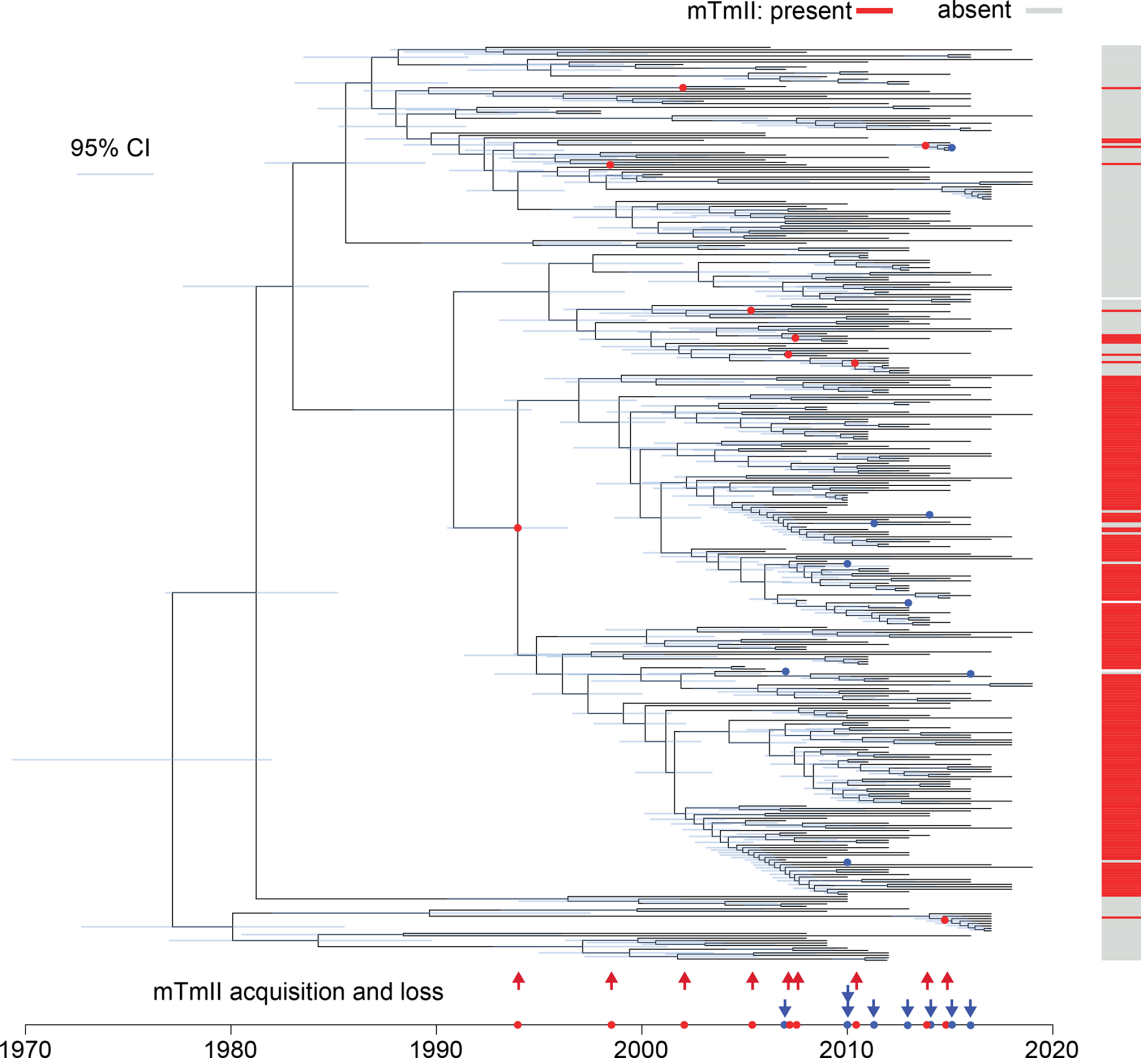
Time-scaled maximum credibility tree of monophasic *S*. Typhimurium ST34 clade and inference of horizontal gene transfer. Dated phylogenetic tree inferred from root-to-tip accumulation of SNPs based on the ML phylogenetic tree of 374 globally distributed monophasic ST34 isolates. Nine independent acquisitions (red arrows and circles) and eight independent losses (blue arrows and circles) of prophage mTmII based on the maximum parsimony principle are indicated.

To investigate the evolutionary process further a temporally structured phylogenetic tree was reconstructed using 5274 core-genome SNP sites from the globally sourced collection of 374 monophasic ST34 isolates. Linear-regression analysis indicated a statistically significant relationship in the root-to-tip accumulation of SNP distance over time (*P*=1×10^−4^, R^2^=0.3, Fig. S5). The common ancestor of the ST34 clade that was estimated to have existed around 1976 (CI95 %=1969–1982) based on this dataset (Fig. 6). The earliest acquisition of prophage mTmII was estimated to be around 1994 (CI95 %=1991–1996) that gave rise to a large subclade with occasional sporadic loss of the prophage. An additional eight acquisitions of prophage mTmII occurred between around 1998 and 2015 (Fig. 6).

## Discussion

Differential sensitivity of *S*. Typhimurium isolates to a panel of phage lysates that define phage type has been used for surveillance in many countries around the world for over 50 years [69]. Geographically and temporally clustered isolates of the same phage type signified potential outbreaks and longer-term prevalence provided trends on the epidemiology of epidemic clones [19, 23, 24]. The identification of outbreaks was effective because the rate of phage-type switching was not great enough over short periods of time. However, the interpretation of epidemiological trends over extended periods using phage-type data depends on stability of the phage type and is therefore prone to inaccuracy if phage-type switching occurs at a high rate. We found considerable variation in the frequency of the dominant phage type within second-order clades that corresponded to recently expanded epidemic clades of *S*. Typhimurium. In most subclades 25–75 % of isolates exhibited the cladal majority phage type, highlighting relatively frequent changes in sensitivity to the phage used in the typing scheme. Subclade 3 (Fig. S1) corresponded to an epidemic clone that emerged around the year 1980 in Europe characterized by the presence of the *sopE* virulence gene on a prophage and phage types 29, 44, 49, 204b and 204 c [19, 70]. In the current dataset, this clade consisted of isolates of which approximately 73 % were of phage type U302, which is resistant to all standard typing phage. This pattern of phage sensitivity represents increased resistance to phage (*Ri*) than DT204/DT49 and was not a phage type associated with this clone during the period is was dominant in Europe, suggesting considerable evolution and change in sensitivity to phage in this population. Subclade 21 (Fig. S1) that corresponded to a pandemic clone that emerged around the year 1990 composed of over 80 % DT104 isolates. The currently dominant pandemic monophasic *S*. Typhimurium ST34 clone (subclade 12 in Fig. S1) had a diverse repertoire of phage types as previously reported [28], despite this subclade forming from a relatively recent clonal expansion [2, 71]. The most common phage type was represented by just 65 % of isolates in this subclade. These findings highlight the limitations of surveillance using phage typing and the advantages offered by whole-genome-sequence-based systems such as those now being used by UKHSA [72].

The Anderson phage-type scheme is informative for surveillance and epidemiological studies because of the diversity of phage-sensitivity profiles exhibited by *S*. Typhimurium isolates. However, the basis of the lytic activity of the phage preparations that results in diverse sensitivity profiles is not known. Multiple phage are present in the typing scheme [69, 73] and seven distinct *Eco*RI restriction patterns were detected in a later analysis of 20 of the 32 phage preparations of the Anderson-typing scheme [74]. It is also known that for some phage preparations adaptated following propagation on one of several strains of *S*. Typhimurium contributed to changes in the lytic activity by modification of the DNA of the original phage through methylation, recombination of the original phage with lysogenic phage of the propagating strain, or activation of lysogenic phage from the propagating strain [74]. Sequence analysis of phage preparations used in the typing scheme is therefore needed to fully define the phage diversity. Nonetheless, the typing phage preparations represent a broad spectrum of activity in lysing genotypically diverse *S*. Typhimurium strains and phage-type data from UKHSA surveillance is the most comprehensive database of phage sensitivity data currently available.

Resistance to phage predation is likely to be a strongly selected phenotype, and consistent with this hypothesis, we found that resistance to all standard typing phage preparations (DT193) was the most widely distributed and polyphyletic phage type. Yet we found that sensitivity to many phages of the Anderson phage-typing scheme is widespread for reasons that are not fully understood. Several *S.* Typhimurium lineages were dominated by isolates that were sensitive to over half of the phages in the Anderson-typing scheme (Fig. 1c). One possibility is that strains from different lineages were not subjected to the same level of selection for resistance due to the range of hosts and environments in which they circulate. Consistent with this idea, we found variability in the level of resistance to phage preparations in the Anderson scheme in the high-order clades α and β that are distinct from one another in the host range of strains [1, 23]. Clade α contains multiple lineages associated with strains circulating in multiple host species, primarily in livestock, while clade β contain primarily host-restricted lineages, often avian species [1, 23]. For example, clade β3 is only isolated from pigeons [75, 76] and was sensitive to all but one of the typing phages [76, 77]. Strains of clade β5 are also highly host adapted, commonly associated with passerine bird species [78], and strains were sensitive to over half of the phage preparations. Our study raises the possibility that host range may affect the diversity or range of phage encountered by bacteria and affecting selection for resistance. There is a need to understand the diversity of phage in animal hosts as this has important implications for the potential use of phage as antimicrobials to control *Salmonella* in livestock and the food chain [79].

The epidemiological record of *S*. Typhimurium from human infections in Europe since the 1960s indicates successive waves of dominant multidrug-resistant clones [23, 80]. Selection for antibiotic resistance or immune evasion cannot explain the emergence and replacement of previously dominant clones since they each have similar repertoires of resistance genes and share immune dominant antigens. This study highlights that increased resistance to phage predation is one possible factor in the clonal replacement. We found that the emergence of the most recent dominant clone, monophasic *S*. Typhimurium ST34, was accompanied by an increase in resistance to phage. Isolates of this clade from 2014 and 2015 were predominantly DT193 characterized by resistance to all phage preparations in the standard Anderson-typing scheme. A significant proportion were of DT120, characterised by resistance to fewer typing phages. and was likely to have been the pattern of sensitivity of the ancestor of the epidemic clade. Increased resistance to phage in DT193 typed strains was mainly through the acquisition of the prophage mTmII, that resulted in increased clonal expansion, supporting the idea that resistance to phage may at least contribute to the success of the clone. Indeed, we found that a trend to increased resistance to typing phage in the Anderson typing scheme for each successive dominant clone observed over the past several decades, DT9, DT204, DT104, DT193. Together these data are consistent with the hypothesis that a high level of resistance is important to enable them to circulate in multiple species of livestock, and furthermore that increased resistance may contribute to replacement of previously dominant epidemic clones.

External factors such as changes in animal husbandry of livestock may also play a role in the survival of *S*. Typhimurium strains with a distinct genotype and therefore contribute to clonal replacement of *S*. Typhimurium observed in the epidemiological record [80]. Between 1995 and 2005, a marked change in the pattern of prevalent *S*. Typhimurium phage types in UK pigs was noted, characterized by a decline in previously dominant types [81], and subsequent emergence of *S*. Typhimurium U288 [82] and monophasic *S*. Typhimurium ST34 [28]. Several changes in animal husbandry on pig farms coincided with this period including bans on meat and bone meal in feed (1994), on stall and tethering practices (1999), and on the use of antimicrobials as growth promotors (2005). Dietary changes including increased reliance on copper and zinc as antimicrobials for growth promotion and potential changes in transmission between animals from housing may have selected distinct genotypes. Phage-mediated transduction or lysogeny is perhaps the most important mechanism providing genotypic variation within bacterial populations [83], exceeding that of other mobile genetic elements [12], and may be indispensable for the survival of microbial populations [84]. The emergence of the monophasic *S*. Typhimurium ST34 epidemic clone was accompanied by multiple horizontal gene-transfer events including the acquisition of a genetic island encoding for multidrug resistance [85], SGI-4, an integrative conjugative element encoding copper metal ion tolerance [27] and prophage mTmV encoding a virulence gene, *sopE,* involved in enteropathogenesis [2]. The acquisition of each of these mobile genetic elements was followed by increased clonal expansion either immediately prior to or since initial emergence of the epidemic clone [2]. Yet lysogeny by additional prophage such as mTmII confers increased resistance to infection by related phage, thereby potentially decreasing horizontal transfer. Our data therefore raises the intriguing possibility that increased resistance to phage observed in epidemic clones of *S*. Typhimurium in livestock may reduce the rate of horizontal gene transfer mediated by phage transduction, thereby limiting the potential for further adaption to changes in the environment. Increased resistance to phage may therefore be a short-term advantage associated with the clonal expansion into newly emerged niches, but may have long-term consequences for future adaptation thereby favouring the emergence of successive clones into a new niche as observed in the epidemiological record [25].

## Supplementary Data

Supplementary material 1

Supplementary material 2

Supplementary material 3

Supplementary material 4

Supplementary material 5

Supplementary material 6
