# Supplementary material for: Increased phage resistance through lysogenic conversion accompanying emergence of monophasic Salmonella Typhimurium ST34 pandemic strain
Source: Microb Genom. 2022 Nov 16;8(11):mgen000897. doi: 10.1099/mgen.0.000897 (PMC9836087; doi:10.1099/mgen.0.000897)
Supplement: Supplementary material 1 [file mgen-8-897-s002.pdf]

## Supplementary Material

# Increased phage resistance through lysogenic conversion accompanying emergence of monophasic *Salmonella* Typhimurium ST34 pandemic strain

Oliver J. Charity[1,5], Luke Acton[1,5], Matt Bawn[1,4], Eleonora Tassinari[1,5], Gaetan Thilliez[1], Marie A. Chattaway[2], Timothy J. Dallman[2], Liljana Petrovska[3] and Robert A. Kingsley[1,5*]

[1] Quadram Institute Bioscience, Norwich Research Park, Norwich, NR4 7UQ

[2] Gastrointestinal Bacteria Reference Unit, UK Health Security Agency (UKHSA), London, United Kingdom, NW9 5EQ

[3] Animal & Plant Health Agency (APHA), Weybridge, London, United Kingdom, KT15 3NB

[4] Earlham Institute, Norwich, NR4 7UZ

[5] University of East Anglia, Norwich NR4 7TJ

*Correspondence to rob.kingsley@quadram.ac.uk

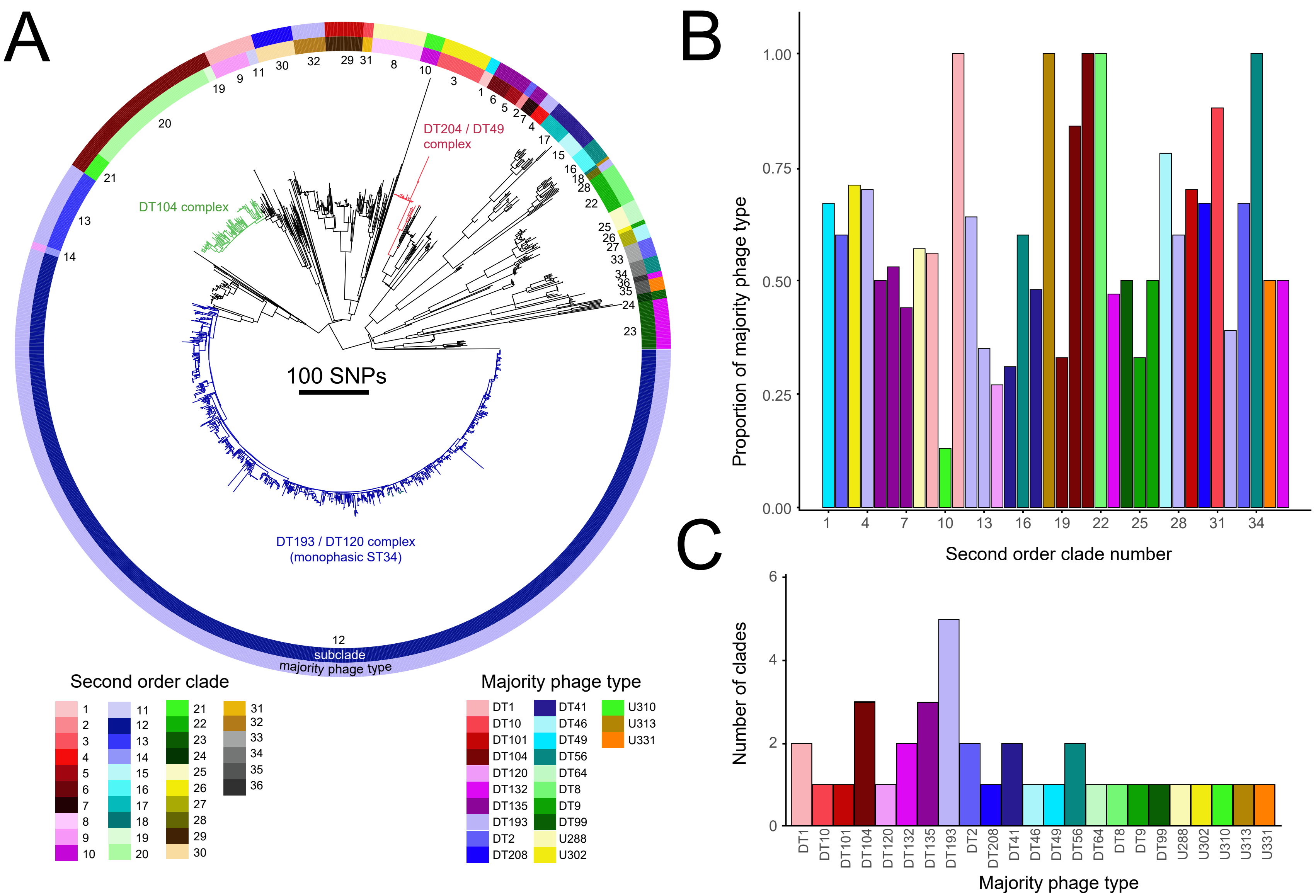


**Supplementary Figure 1. Identification of majority phage type and frequency in major clades of *S.* Typhimurium ST34.** (A) Maximum-likelihood tree based on variation of 16,681 core genome SNP sites from 1,413 whole genome sequences of monophasic *S.* Typhimurium ST34 isolates. (B) The proportion of isolates in each second order clade with the majority phage type (bars). (C) The number of clades with each majority phage type (bars).

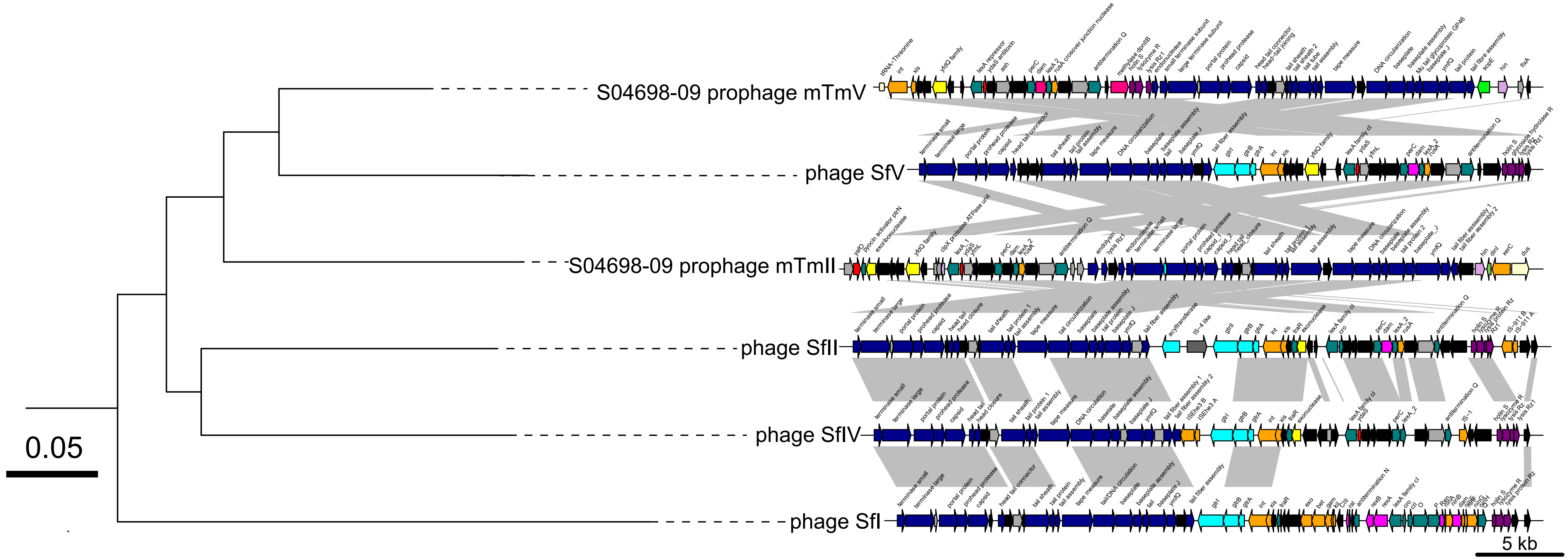


**Supplementary Figure 2. Phylogenetic relationship and sequence similarity of S04698-09 prophage mTmII and related phage.** Phylogenetic relationship and linear genetic diagrams showing the position, size, and orientation of genes for phages similar to prophage mTmII. The neighbour joining phylogenetic tree was constructed using open reading frame (ORF) translated amino acid sequences. ORFs are colour coded by function as follows: hypothetical attB sites (white); integration and excision including IS elements (orange); phage lysogeny regulation (dark green); nucleases (yellow); phage defence (bright pink); a gene encoding virulence factor SopE (bright green); phage particle structure and assembly (dark blue); glucosyl and acyl transferases (bright blue); hypothetical toxin-antitoxins (red); host cell lysis (dark purple); phage associated hypothetical proteins (grey), and; other hypotheticals (black).

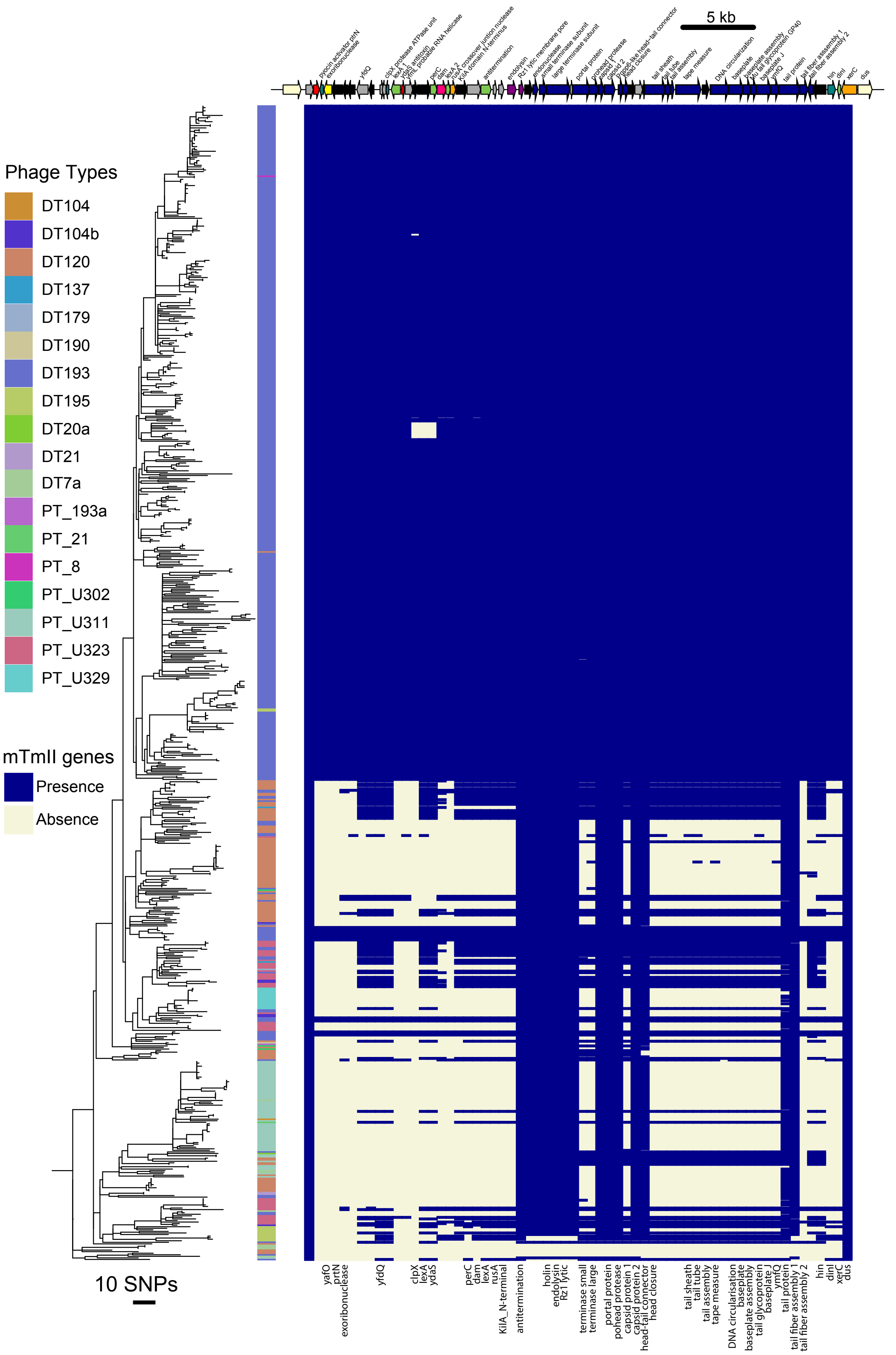


**Supplementary Figure 3. Presence of genes of mTmII in monophasic *S*. Typhimurium ST34 isolates from human infections in England and Wales 2014-2015.** Maximum likelihood tree based on variation in the core genome sequence of 723 monophasic *S*. Typhimurium ST34 isolates. Presence of sequence reads mapping to each gene in mTmII with >90% sequence identity are indicated in the heat map.

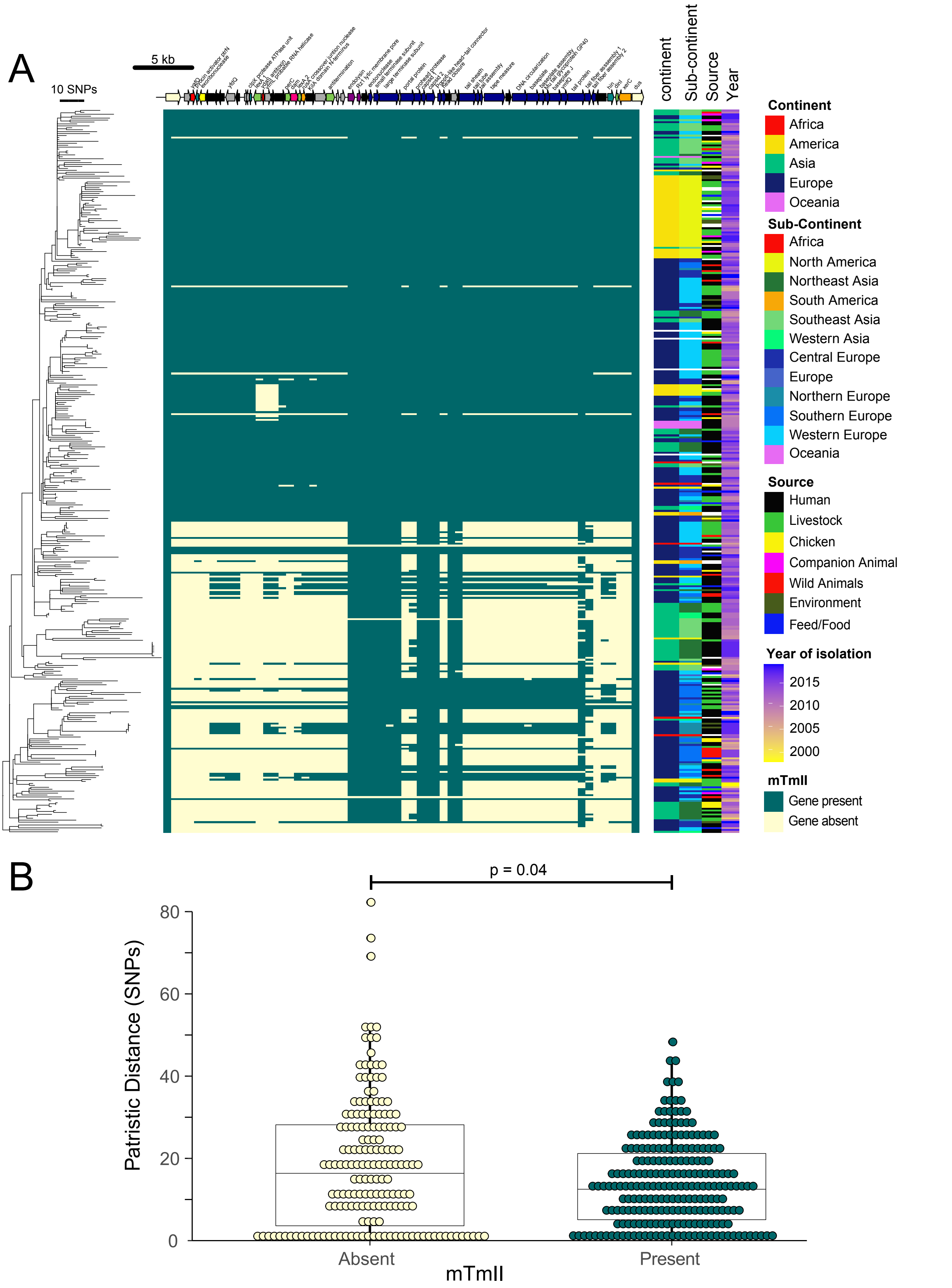


**Supplementary Figure 4. Presence of genes of mTmII in monophasic *S*. Typhimurium ST34 isolates from various sources from geographical locations worldwide.** Maximum likelihood tree based on variation in the core genome sequence of 374 monophasic *S*. Typhimurium ST34 isolates. Presence of sequence reads mapping to each gene in mTmII with >90% sequence identity are indicated in the heat map.

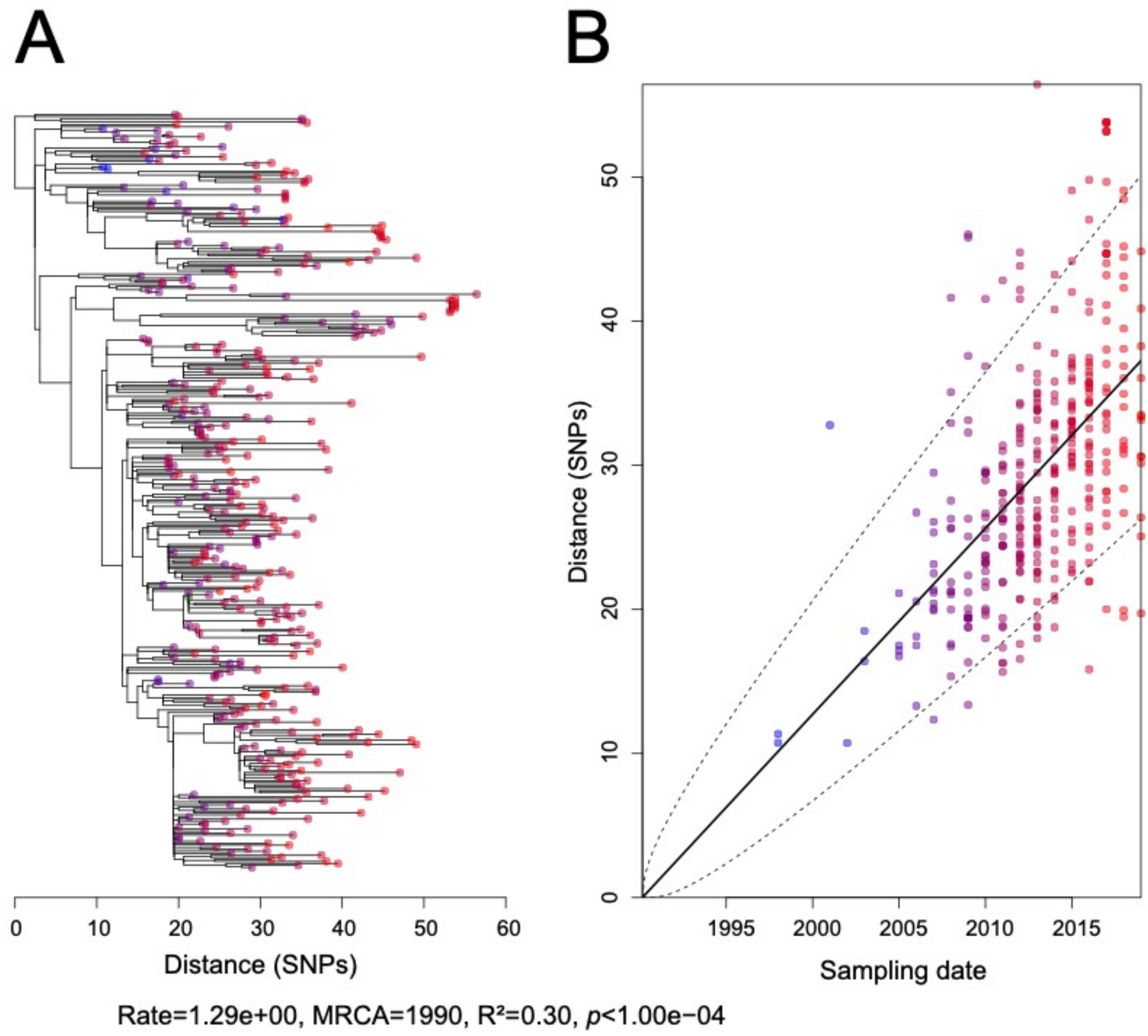


**Supplementary Figure 5. Root to tip analysis of SNPs in monophasic *S*. Typhimurium ST34 and calculation of molecular clock rate.** (A) Maximum-likelihood phylogenetic tree constructed using 3,826 core genome SNP sites from 427 monophasic *S.* Typhimurium ST34 strains showing core genome SNP distance from root to tip. (B) Least squares regression of core genome SNP distance and strain sampling date (solid line) displaying 95% confidence intervals (dashed lines).
